# Understanding the role of oral and vaginal microbiomes in HPV-related cervical, head, and neck cancers: knowledge gaps and feasibility in Sub-Saharan Africa

**DOI:** 10.3389/frmbi.2025.1576394

**Published:** 2025-12-05

**Authors:** Hélène Eya Kamassa, Gnatoulma Katawa, Abiola Isawumi, Charles Olwal, Winfried Seth Gbewonyo, Peter Kojo Quashie, Yaw Bediako

**Affiliations:** 1 West African Centre for Cell Biology of Infectious Pathogens (WACCBIP), University of Ghana, Accra, Ghana; 2 Department of Biochemistry, Cell and Molecular Biology, College of Basic and Applied Sciences, University of Ghana, Accra, Ghana; 3 Laboratoire de Microbiologie et de Contrôle de Qualité des Denrées Alimentaires, Unité de Recherche en Immunologie et Immunomodulation (UR2IM), Ecole Supérieure des Techniques Biologiques et Alimentaires (ESTBA), Université de Lomé, Lomé, Togo; 4 Yemaachi Biotech, Accra, Ghana

**Keywords:** microbiome dysbiosis, vaginal microbiota, oral microbiota, HPV, immune evasion, cervical/head and neck cancers, sub-Saharan Africa

## Abstract

Microbiome dysbiosis, characterized by an imbalance in the composition of microbial communities, has emerged as a potential risk factor for the development of cervical, head, and neck cancers. While previous studies have predominantly focused on high-income countries, there is a significant gap in understanding the relationship between microbiome alterations and cancer development in sub-Saharan Africa. Considering the unique socio-economic and environmental factors in this region, investigating the role of vaginal and oral microbiota in the progression of these cancers is crucial. This review explores the involvement of microbial dysbiosis in cervical, head, and neck cancers, particularly how it influences Human Papillomavirus-driven immune evasion, and highlights the importance of microbiota profiling in sub-Saharan Africa. The implications of these insights for cancer prevention and treatment strategies in this population are also discussed.

## 1 Introduction

Cervical cancer (CC) is ranked as the fourth most commonly diagnosed cancer and the fourth leading cause of cancer-related deaths among women, with an estimated 604,000 new cases and 342,000 deaths yearly worldwide. The majority of affected countries are located in sub-Saharan Africa, Melanesia, South America, and South-Eastern Asia. Similarly, oral squamous cell carcinoma (OSCC) is the most prevalent subgroup of head and neck cancer and represents a major cause of morbidity and mortality worldwide. Head and neck cancers were identified as the seventh most common cancer worldwide in 2018, with 378,000 new cases in 2024 and 178,000 reported deaths (Sung et al., 2021). Sub-Saharan Africa faces a high burden of cervical, head, and neck cancer, with limited resources for early detection and treatment (Atnafu et al., 2024). Sub-Saharan Africa faces a high burden of cervical, head, and neck cancer, with limited resources for early detection and treatment (Atnafu et al., 2024).

High-risk Human Papillomavirus (Hr-HPV) is widely recognized as the primary cause of cervical cancer. However, in recent years, growing evidence indicates its involvement in other types of malignancies. Specifically, HPV has been identified as a contributing factor in a subset of head and neck cancers, highlighting its pathogenic role beyond cervical region (Sabatini and Chiocca, 2020). About 40 genotypes of HPVs have been identified and categorized as low, medium, or high-risk depending on their clinical oncogenicity. Low-risk HPVs cause benign lesions, while high-risk HPVs are associated with premalignant and malignant lesions. However, the prevalence and distribution of HPV genotypes vary considerably across different regions (Kuassi-Kpede et al., 2021).

Most sexually active women contract at least one high-risk genital HPV type during their lifetime, but only a small fraction will progress to cervical cancer (Adebamowo et al., 2017). The variability in cervical cancer progression has been attributed to host immune responses and cervicovaginal dysbiosis, underscoring the involvement of multiple cofactors in the disease’s pathogenesis (Reimers et al., 2016). Notably, studies have highlighted an association between cervicovaginal dysbiosis and cervical intraepithelial neoplasia, a precursor to cervical cancer (Marrs et al., 2012; Torcia, 2019; Zhang et al., 2021). Similarly, emerging evidence suggests that oral microbiota dysbiosis may play a role in the development of head and neck cancers, particularly in HPV-driven cases, highlighting the critical importance of microbiota composition in modulating cancer risk and progression across anatomical sites (Benjamin et al., 2023; Constantin et al., 2023).

Emerging evidence suggests that the oral and vaginal microbiomes may share immunological and microbial communication pathways that influence HPV persistence and oncogenic progression. Understanding these potential cross-mucosal interactions could provide a more integrated view of HPV-associated carcinogenesis. For instance, research has shown that vaginal dysbiosis can trigger chronic inflammation and alter the host immune response, leading to increased susceptibility to cervical cancer (Kumari and Bhor, 2021; Zhou et al., 2021; Di Tucci et al., 2023). Furthermore, dysbiosis has also been shown to affect the expression of genes that regulate key processes in cancer progression, such as cell proliferation, angiogenesis, and invasion (Liu et al., 2020; Akbar et al., 2022).

Bacterial vaginosis is the main vaginal microenvironment disorder reported in sub-Saharan Africa (Tchelougou et al., 2013; Katawa et al., 2021). However, most of the studies characterizing the vaginal microbiome are light microscopy and culture-based. Metagenomic sequencing offers a powerful tool to reveal community structures and their gene functions at a far greater resolution than culture-based methods (Deceuninck et al., 2000; Lassey et al., 2005; Konadu et al., 2019).

This review aims to synthesize current knowledge on the potential interactions between these microbiomes and cancers, highlight the importance of microbiota profiling in a high-risk region, and suggest directions for future research and clinical applications.

## 2 HPV: genome organization and types distribution

**Figure 1:**
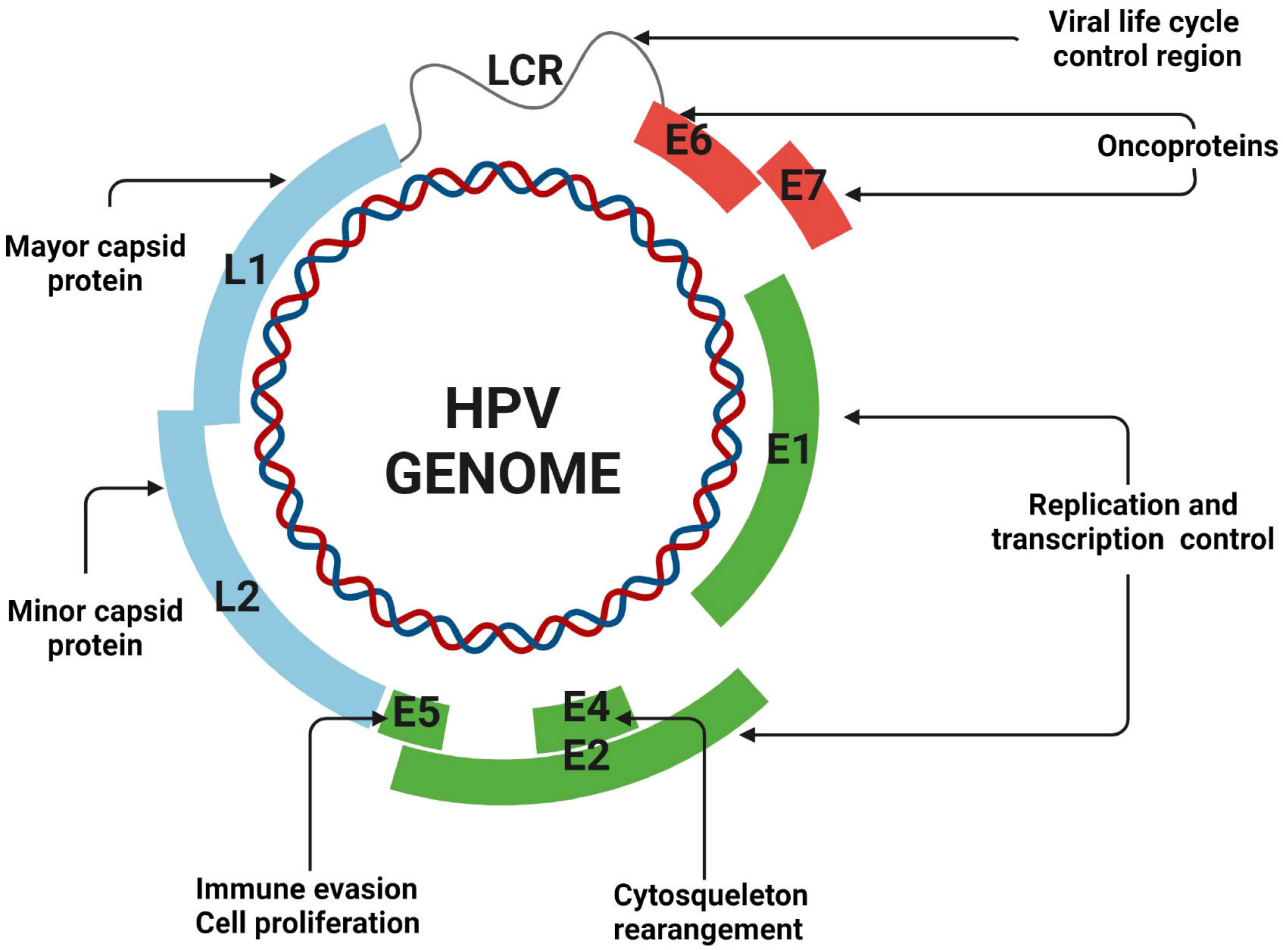
Genome structure of human papillomavirus. All HPV genome consists of circular double-stranded DNA categorized into three main regions: the early region (E), the late region (L), and the long control region (LCR). The LCR is responsible for regulating the viral life cycle. The gene product of the early region encodes proteins E1, E2, E4, E5, E6, and E7, which are involved in replication, transcription control, cytoskeletal rearrangement, immune evasion, and cell proliferation. E6 and E7 are oncogenic proteins that disrupt cell cycle regulation and contribute to malignancy. The late region encodes structural proteins L1 and L2, which form the major and minor capsid proteins, essential for viral assembly and infection. Generated in biorender.

Human papillomaviruses (HPVs) are small non-enveloped viruses of approximately 55 nm diameter. Their genome consists of a circular double-stranded DNA molecule of 8 kb in length (zur Hausen, 2009). The genome is broadly divided into three regions: early, late, and long control region. The early region encodes non-structural proteins (E1, E2, E4 to E6 and E7). E1 and E2 gene products regulate the viral life cycle from replication to transcription, E4 regulate cytoskeleton rearrangements while E6 and E7 cause cell-cycle deregulation. The late region codes for the L1 and L2 capsid proteins which form the structure of the virion. Although there is significant sequence variation between various forms of HPV, the architecture of the genome is substantially conserved within each type (**Figure 1**) (Blanco et al., 2021).

About 200 different types of HPV have been classified as alpha, beta, gamma, delta and mu genera based on their nucleotide sequences variation. The oncogenic HPV types associated with cervical cancer belong to ‘Alpha’ genus (Ibeanu, 2011). HPV ‘variants’ differ generally by <3% in their L1 sequences, and mostly exhibit single-nucleotide variation. HPV types are tissue tropic, and mostly lesion specific (Pal and Kundu, 2019). About, 30–40 genotypes of HPV are known to infect the anogenital and the cervicovaginal mucosa of human either during sexual intercourse or just via skin contact of the genitals of human. They are categorized into 3 groups according to their clinical oncogenic potential: non-oncogenic or low risk-HPV types (HPV 6, 11, 40, 42, 54, 55, 61, 62, 64, 67, 69, 70, 71, 72, 81, 83, 84 and 89), probable oncogenic types or moderate risk (HPV 26, 53, and 66) and oncogenic types or high-risk HPV (HPV 16, 18, 31, 33, 35, 39, 45, 51, 52, 56, 58, 59, 68, 73, and 82) (de Villiers, 2013). However, there is considerable regional variation in sub-Saharan Africa (SSA), with the highest incidence of HPV infection and cervical cancer observed in Eastern and Western Africa (De Vuyst et al., 2013). Furthermore, HPV genotypes are unevenly distributed in the region, hence the necessity to conduct systematic operational studies for periodic updates on the virulence of these genotypes. In SSA, several studies depicted distinct genotypes involved in cervical lesions. For instance, in West Africa, HPV genotypes 18 and 16 predominated in Cameroon (Tchouaket et al., 2022), whereas HPV 58 exhibited the highest prevalence in the central region of Togo, and HPV 31 was notably prevalent in southern Togo (Kuassi-Kpede et al., 2021; Omondi et al., 2022). In Benin, HPV genotype 59 was prominently represented (Piras et al., 2011). Furthermore, in Ghana, elevated frequencies of HPV 16 and HPV 18 were detected in the Accra metropolitan area and Kumasi (Nartey et al., 2023).

## 3 Cervical cells organization

The lowest part of the uterus, or womb, is referred to anatomically as the cervix. Squamous and columnar epithelial cells constitute the structure of cervix. Squamous epithelial cells form the cervical epithelium’s layers while columnar cells form the glandular epithelium of the cervix. The junction where these two layers meet is the squamo-columnar junction or the transformation zone (TZ) (Doorbar and Griffin, 2019). TZ is the area where columnar epithelium transforms into squamous epithelium during embryological cell differentiation. The first stage of carcinoma process termed Dysplasia, starts at this junction. Cervical cancer can be divided into two major types according to its epithelial origin. Dysplasia originating from cervical squamous cells is called cervical squamous cell carcinoma (SCC) while the one from glandular epithelial cells is called cervical adenocarcinoma (ADC) (Hopman and Ramaekers, 2017). It has been reported that the intact cervical epithelium is resistant to viral infections. However, HPV entry occurs when this epithelium’s integrity is compromised chemically or mechanically. For instance, severe micro-abrasions that occur during sexual activity facilitate HPV infection in naive basal layer cells (Norenhag et al., 2020).

## 4 HPV replication cycle

HPV enters naive basal epithelial cells of the cervix via binding of L1 to heparin sulfate proteoglycan receptors in the epithelial basal membrane. The virus capsid then undergoes a conformational change, allowing the exposure of L2. The newly exposed site on L2 binds to surface molecules on the wound keratinocyte to complete viral entry. Virion undergoes endosomal transport, uncoating, and cellular sorting after internalization. While the L1 protein is kept in the endosome and later degraded by the lysosome, the L2 protein-DNA complex ensures that the viral genomes enter the nucleus where viral transcription initiates (Ozbun and Campos, 2021).

As basal cells progress through maturation and reach the terminally differentiated epithelial layer, the expression of L1 and L2 capsid proteins increases, facilitating the assembly of viral particles. These particles, along with the shed squamous cells, contribute to the ongoing transmission and infection of the virus (Doorbar et al., 2012). This sequence represents the prototypical replication cycle observed across various HPV genotypes. HPV encodes only one DNA replication enzyme, E1. The viral E2 protein’s replication ultimately depends on the host DNA synthetic machinery. Nonetheless, E6 and E7 gene products initiate cellular DNA synthesis in non-cycling cells, inhibit apoptosis, and delay the differentiation program of the infected keratinocyte, creating a favorable environment for viral DNA replication in the non-cycling cells (Doorbar et al., 2012; Pal and Kundu, 2019).

In the cervical epithelium, basal cells reside adjacent to the basement membrane, adhering to the extracellular matrix (ECM). Studies indicate that as basal cells progress toward the epithelial surface, growth signals are halted, and differentiation of the epithelium predominates. This regulatory transition is crucial, and its disturbance is recognized as one mechanism by which E6 oncogene induces cellular transformation. The attachment of basal cells to the ECM involves adhesion molecules like paxillin and zyxin (Kombe Kombe et al., 2020). Interactions with E6 disrupt normal cell adhesion and signaling pathways, leading to epithelial cell detachment from the extracellular matrix. In continually dividing epithelial cells, both the inhibition of terminal differentiation and the preservation of the viral replication milieu support ongoing viral replication (Ibeanu, 2011).

## 5 Cervical cancer pathogenesis

The E6 and E7 proteins, which have been linked to several modulatory roles, are the main transforming proteins of high-risk HPV strains. The tumor suppressor p53 and the retinoblastoma protein (Rb) are two of E6 and E7’s main targets. E6 from high-risk HPV strains not only targets p53 for degradation but also activates telomerase (Huibregtse and Beaudenon, 1996). Mechanical studies have shown the key process by which E6 immortalizes epithelial cells through telomerase activity. It has been demonstrated that high-risk HPV type E7 activates telomerase by disrupting the retinoblastoma protein (Rb)/p16 pathway while E6 binding to E6AP and triggers hTERT (human telomerase reverse transcriptase) (Oh et al., 2001). This process also relies on the c-Myc oncogene, which acts on the hTERT promoter to increase its transcription. Furthermore, it appears that E6-E6AP binding raises hTERT activity *in vivo* via interacting with two NFX-1 isoforms, a gene repressor that binds to the X1 box. While binding to NFX1–123 directly induces hTERT promoter activity, ubiquitination of NFX1–91 prevents the hTERT promoter from being repressed (Katzenellenbogen et al., 2009).

Concomitantly, E7 oncoprotein from high-risk human papillomaviruses (HPVs) not only binds to but also modifies the activity of key cell cycle regulatory proteins, including members of the retinoblastoma (Rb) protein family and histone deacetylases (HDACs). Indeed, E7 interacts with histone deacetylases (HDACs), leading to enhanced E2F-driven transcription and facilitation of S phase progression in epithelial cells. Beyond its role in cell cycle disruption, E7 orchestrates diverse structural alterations in the genomic landscape of epithelial cells, including the formation of extra chromosomal material, polar mitoses, anaphase bridging, and the induction of aneuploidy (Longworth and Laimins, 2004). For instance, the high-risk HPV oncoproteins E6 and E7 collaborate to induce anomalies in centrosome numbers, irregular formation of mitotic spindle poles, and genomic instability. In contrast, the low-risk HPV-6 E6 and E7 proteins do not elicit these aberrations (Duensing et al., 2000).

The majority of women encounter HPV virus at least once in their lifetime but the infection persists in a minority and progresses to cervical cancer. Typically, HPV infections tend to regress within 12 months. However, the transition from initial HPV infection to the development of carcinogenesis typically takes an average lag time of 10 to 20 years. This observation suggests the presence of other potential cofactors that play a role in determining the latency, regression, or progression of HPV infections over time. These factors may contribute to the variability in outcomes seen among individuals infected with HPV.

## 6 Head and neck cancers pathogenesis

Head and neck cancer refer to a group of cancers that develop in the tissues and structures of the head and neck region. This area includes the oral cavity (lips, tongue, gums, inside of the cheeks), throat (pharynx), voice box (larynx), salivary glands, nasal cavity, and sinuses (Owens et al., 2022). There are various types of head and neck cancers, including squamous cell carcinoma Oral Squamous Cell Carcinoma (SCC), which is the most common type, as well as cancers of the mouth, tongue, throat, voice box, and nasal cavity (Johnson et al., 2020). These cancers usually arise from cells lining the mucosal surfaces of these areas. Risk factors for head and neck cancer include tobacco and alcohol use, as well as certain viral infections like human papillomavirus (HPV) and Epstein-Barr virus (EBV). Exposure to certain chemicals, such as asbestos and formaldehyde, and a weakened immune system are known risk factors for this cancer (Dhull et al., 2018). Symptoms may vary depending on the location and stage of the cancer, but common signs include persistent pain or a sore throat, difficulty swallowing or speaking, a lump or swelling in the neck, changes in voice, ear pain, and unexplained weight loss.

Genetic abnormalities and dysregulated protein expression are hallmarks of the intricate, multi-step molecular pathogenesis of head and neck cancer, changes in important regulatory proteins like EGFR and p53 are some of the most common molecular processes that drive transformation (Park et al., 2010). Dysfunction of the tumor suppressor gene p53, often through mutation, results in a loss of normal growth control mechanisms. This leads to uncontrolled cell proliferation, increased survival, enhanced migratory potential, and promotion of angiogenesis, all of which contribute to tumor development and progression (Li et al., 2023). Similarly, alterations in the epidermal growth factor receptor (EGFR) pathway play a significant role in the molecular pathogenesis of head and neck cancer. Aberrant EGFR signaling, often due to gene amplification or overexpression, results in increased cell proliferation, survival, migration, and angiogenesis, further driving tumor growth and metastasis (Psyrri et al., 2013; Santi et al., 2023).

Moreover, the tumor microenvironment surrounding head and neck cancers plays a critical role in facilitating tumor progression. This microenvironment consists of various cellular and non-cellular components, including immune cells, fibroblasts, blood vessels, extracellular matrix proteins, and signaling molecules (Wei et al., 2020). Interactions between tumor cells and these components contribute to the development of a supportive niche for tumor growth, invasion, and metastasis. Genetic alterations in precancerous cells within the head and neck region are pivotal in initiating the transformation process, ultimately leading to tumor development. These alterations, which can include mutations, amplifications, deletions, and epigenetic modifications, disrupt the normal regulatory mechanisms of cell growth, division, and differentiation. As these genetic aberrations accumulate over time, they create a favorable environment conducive to tumorigenesis, where the affected cells gain a survival advantage and proliferate uncontrollably. The dynamic interplay between molecular alterations within tumor cells and compensatory changes in the tumor microenvironment is key to the aggressive nature of head and neck cancers. For example, tumor cells may secrete factors such as TGFb, IL10, CCL2, myeloid-derived suppressor cells (MDSCs), vascular endothelial growth factor (VEGF), PD-L1 and Matrix Metalloproteinases (MMPs) that recruit immune cells and promote an immunosuppressive environment, allowing them to evade immune surveillance and facilitate metastasis (Umansky et al., 2016). Additionally, tumor-associated fibroblasts and endothelial cells may remodel the extracellular matrix and promote angiogenesis, providing essential nutrients and oxygen to tumor growth (Umansky et al., 2016).

## 7 Immunomodulation and HPV infection

Mammals have developed intricate innate and adaptive immune mechanisms to manage both local and systemic viral infections, minimizing host damage when infections persist. The replication cycle of human papillomavirus (HPV) provides specific survival advantages, enabling the virus to persist in some individuals over extended periods (Bordignon et al., 2017). The sequestration of HPV replication within epithelial cells presents an intriguing phenomenon known for its absence of a bloodborne phase. This unique feature effectively prevents the virus from entering the bloodstream, enabling it to replicate quietly without provoking a strong immune response. By releasing viral particles from the outer layer of epithelial cells through non-destructive processes, HPV avoids triggering inflammatory mediators (Senba and Mori, 2012). Consequently, asymptomatic HPV infections frequently occur in immunocompetent women, even in those harboring high-risk HPV strains, as the virus adeptly operates stealthily within the epithelial microenvironment. This immune evasion strategy underscores the complexity of HPV infection dynamics and highlights the challenges in effectively targeting the virus for therapeutic intervention (Stanley, 2012).

In addition to their role in enhancing cellular transformation, the co-expression of E6 and E7 in high-risk human papillomavirus (HPV) types orchestrates a sophisticated modulation of molecules involved in both the innate and adaptive immune responses. This strategic manipulation potentially enables HPV to evade immune surveillance, particularly during the early stages of replication. The key processes include disruption of innate immune sensing (Westrich et al., 2017), inhibition of antigen presentation (Bashaw et al., 2017), suppression of Major Histocompatibility Complex (MHC) Class I expression (Kim et al., 2011), inhibition of interferon signaling (Scott et al., 2020), and consequently modulation of cytokine expression, immune suppression and immune tolerance (Torres-Poveda et al., 2014).

**Figure 2:**
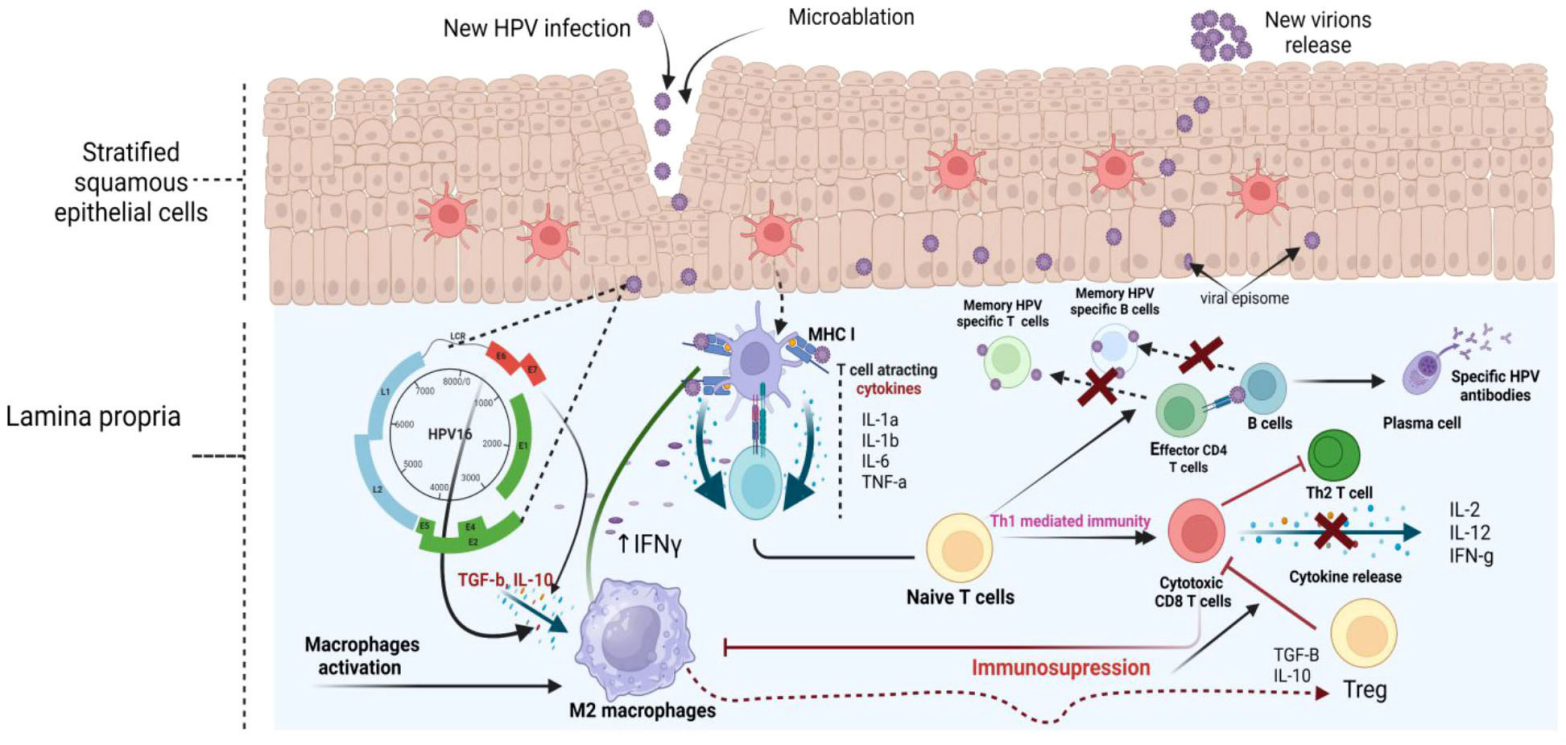
Immune evasion mechanisms in HPV infection and their impact on immune memory. Upon entering through micro-abrasions, the virus infects basal cells, initiating its replication cycle and releasing new virions in the upper epithelial layers. In the lamina propria, macrophages and dendritic cells typically recognize HPV antigens to activate the immune system. However, HPV manipulates this response by inducing the release of immunosuppressive cytokines such as TGF-β and IL-10, which polarize macrophages toward the anti-inflammatory M2 phenotype. This shift suppresses MI macrophages and weakens the release of pro-inflammatory cytokines (IL-1a, IL-1β, IL-6, TNF-a), impairing the recruitment and activation of cytotoxic CD8+ T cells by downregulating MHC I expression. As a result, the activation of naive T cells into Th1 (cell-mediated immunity) and Th2 (antibody production) pathways is diminished, preventing robust immune memory formation. T regulatory cells further exacerbate immune suppression by releasing TGF-β and IL-10, which inhibit cytotoxic T cells. Additionally, HPV interferes with B cell differentiation into plasma cells, limiting the production of specific HPV antibodies. These combined mechanisms allow HPV to persist in the host, evade immune detection, and increase the risk of cancer development by preventing the formation of effective immune memory. Generated in Biorender.com.

E6 influences the interaction between epithelial cells and antigen-presenting cells, such as Langerhans cells, within the epidermis by modulating the expression of the cell surface protein E-cadherin. Notably, a decrease in Langerhans cell numbers and diminished E-cadherin levels are hallmark features of HPV infection. Under normal conditions, epithelial cells secrete cytokines to induce chemotaxis, facilitating the repopulation of Langerhans’ cell precursors. This chemotactic activity is typically mediated by macrophage inflammatory protein MIP-3alpha. However, in cells exhibiting evidence of E6 and E7 expression, MIP-3alpha expression is inhibited, resulting in impaired antigen presentation (Matthews et al., 2003). Concurrently, E6 exerts its immunomodulatory effects by inhibiting the ability of interferon regulatory factor 3 (IRF3) to induce the activation of interferon beta, thus thwarting the innate immune system’s initial response to viral infection (Ronco et al., 1998). Similarly, E7 disrupts immune signaling by binding to interferon regulatory factor 1 (IRF1), preventing the activation of interferons alpha and beta (Castro-Muñoz et al., 2022). Furthermore, several evidence have demonstrated that the expression of E6 and E7 inhibits toll-like receptor 9 (TLR9), failing in cytokine production (Hasan, 2014). This mechanism further hampers the host immune response against HPV infection. While the precise mechanisms underlying these immune evasion strategies are not yet fully elucidated, their orchestrated interplay highlights the sophisticated tactics employed by HPV to evade immune surveillance and promote oncogenesis. Furthermore, E7 can downregulate the expression of MHC class I molecules on the surface of infected cells (Gameiro et al., 2017). MHC class I molecules play a key role in presenting viral antigens to cytotoxic T lymphocytes (CTLs), which are essential for the recognition and elimination of virus-infected cells. By reducing MHC class, I expression, E7 helps HPV-infected cells evade CTL-mediated immune surveillance. These intricate immune evasion mechanisms highlight the adaptability of HPV and its ability to subvert host immune responses, ultimately promoting persistent infection (**Figure 2**). While HPV has evolved multiple strategies to subvert host immunity, such as downregulating antigen presentation and modulating cytokine signaling, these viral mechanisms operate within a broader ecological context shaped by the local microbiota.

## 8 Mucosal microbiomes in HPV-driven diseases

The human mucosal surfaces, particularly the oral and vaginal cavities, represent critical ecological niches for human papillomavirus (HPV) infection and persistence (Deo and Deshmukh, 2019). These mucosae are not only physical barriers but also dynamic immunological environments shaped by resident microbial communities (Moreno et al., 2023). Dysbiosis, defined as the disruption of the normal microbial balance at either site, can profoundly influence local immune responses, alter epithelial homeostasis, and facilitate viral persistence, thereby contributing to the risk of HPV-associated malignancies (Zaura et al., 2014). Despite the anatomical and functional differences between the oral and vaginal niches, emerging evidence suggests that similar microbial mechanisms, including the depletion of protective commensals and the enrichment of pro-inflammatory taxa, may converge to promote HPV-driven disease. It has been shown that specific microbial communities can modulate local immunity and influence the course of HPV infection. In the vaginal microenvironment, the transition from a *Lactobacillus*-dominated flora to one enriched with *Lactobacillus iners*, *Gardnerella vaginalis*, *Atopobium vaginae*, and *Prevotella* species is associated with epithelial barrier disruption, reduced mucosal integrity, and altered cytokine profiles that favor viral persistence (Amabebe and Anumba, 2018; Constantin et al., 2023). *L. iners*, despite being a *Lactobacillus* species, produces limited lactic acid and hydrogen peroxide, creating a less protective environment that may facilitate HPV survival (Vaneechoutte, 2017; Zheng et al., 2021). Similarly, *G. vaginalis* and other anaerobes contribute to pro-inflammatory states through the production of sialidases and short-chain fatty acids, which can impair epithelial repair mechanisms and enhance viral access to basal cells (Molina et al., 2022; Wu et al., 2022). In the oral cavity, dysbiosis characterized by the overrepresentation of *Fusobacterium nucleatum*, *Porphyromonas gingivalis*, and *Candida albicans* has been linked to chronic inflammation and immune evasion (McIlvanna et al., 2021). *F. nucleatum* and *P. gingivalis* can modulate Toll-like receptor signaling and dampen dendritic cell activation, thereby interfering with antiviral immune surveillance. *C. albicans*, through its capacity to induce Th17-mediated inflammation and oxidative stress, may further potentiate HPV-induced epithelial transformation (Yáñez et al., 2024). These dysbiotic shifts promote a microenvironment conducive to viral oncogene expression (E6/E7) and persistent immune evasion, bridging microbial imbalance with HPV-driven carcinogenic progression across mucosal sites (MacLean et al., 2024). Given the pivotal role of mucosal microbial communities in shaping host immune responses and influencing HPV persistence, the vaginal microbiome offers one of the most well-characterized examples of these interactions.

## 9 Cervicovaginal microbiome

Vaginal microbiota composition is a key component in women’s health and reproduction. It’s a dynamic and complex system regulated by many factors including age, diet, hormonal shift, sexual lifestyle, hygiene practices, use of contraception, hormonal shift, age as well as genetics (Norenhag et al., 2020). In all women, the vaginal mucosal ecosystem consists of stratified squamous epithelium protected by a mucosal layer through cervicovaginal fluid (CVF). This later contains mucins and antimicrobial molecules including b-defensin, Lipocalin, and Elafine as well as antibodies (IgA and IgG) produced by mucosal plasma cells. The homeostasis of the vaginal ecosystem is maintained by CVF and represents the first line of defense against exogenous pathogen colonization through the activity of mucins which entrap microbes and facilitate their binding to the secretory antibodies. CFV also contains the vaginal microbiota, which co-exist in a mutualistic relationship with the host. For instance, lactobacillus creates a low pH environment protecting against both exogenous bacteria and viruses by producing lactic acid, bacteriocins, and biosurfactants (Amabebe and Anumba, 2018). The vaginal microenvironment also includes epithelial cells, bacteria, and of innate and adaptive immune cells including neutrophils, macrophages, classic dendritic cells, Langerhans cells, NK cells, T and B lymphocytes. Dendritic cells and monocyte/macrophages represent the most prevalent antigen-presenting cells in the vaginal ecosystem.

The vaginal microbiota has been categorized in five community stapes types (CSTs), four of which are dominated by single species of Lactobacillus (CST I-*L. crispatus*, CST II-*L. gasseri*, CST III-*L. iners*, CST V-*L. jensenii*) (Norenhag et al., 2020; France et al., 2022). However, there is a remarkable variability in vaginal microbiota among women all over the world. Several studies revealed that ethnicity/race influences the composition of CVMs. For instance, a cross sessional study conducted in American population comparing CVMs of reproductive age women among four different ethnicities (White, Black, Hispanic, and Asian) have shown that most of white and Asian women had Lactobacillus dominated CVMs compared to Black and Hispanic. Other studies also revealed that white and Asian women are more likely to have CVMs dominated by a single or multiple lactobacillus, especially L. crispatus than Black women (Jespers et al., 2012; Borgdorff et al., 2017; Vodstrcil et al., 2017). A comparative study of European and African American reproductive-age women CVMs strongly correlated American from African ancestry to higher relative abundance of non-lactobacillus-dominated microbiotas (Fettweis et al., 2014). Consequently, Vaginal pH was also found to vary by ethnicity; with Black and Hispanic women having higher vaginal pH relative to White and Asian women (Ravel et al., 2011). Another study conducted in north American population reported that the prevalence of non-Lactobacillus-dominated CVMs was about 5 fold change higher among healthy American African women compared to Caucasian women; suggesting that the high prevalence of asymptomatic BV and STI among African women might be due to this variability (Zhou et al., 2007).

Although distinct in anatomy and function, the vaginal and oral microbiomes share key features in their interactions with HPV, including roles in immune modulation and epithelial integrity. Exploring the oral microbiome thus offers complementary insight into how microbial diversity and dysbiosis across mucosal sites may influence HPV persistence and disease progression.

## 10 Oral microbiome

**Figure 3:**
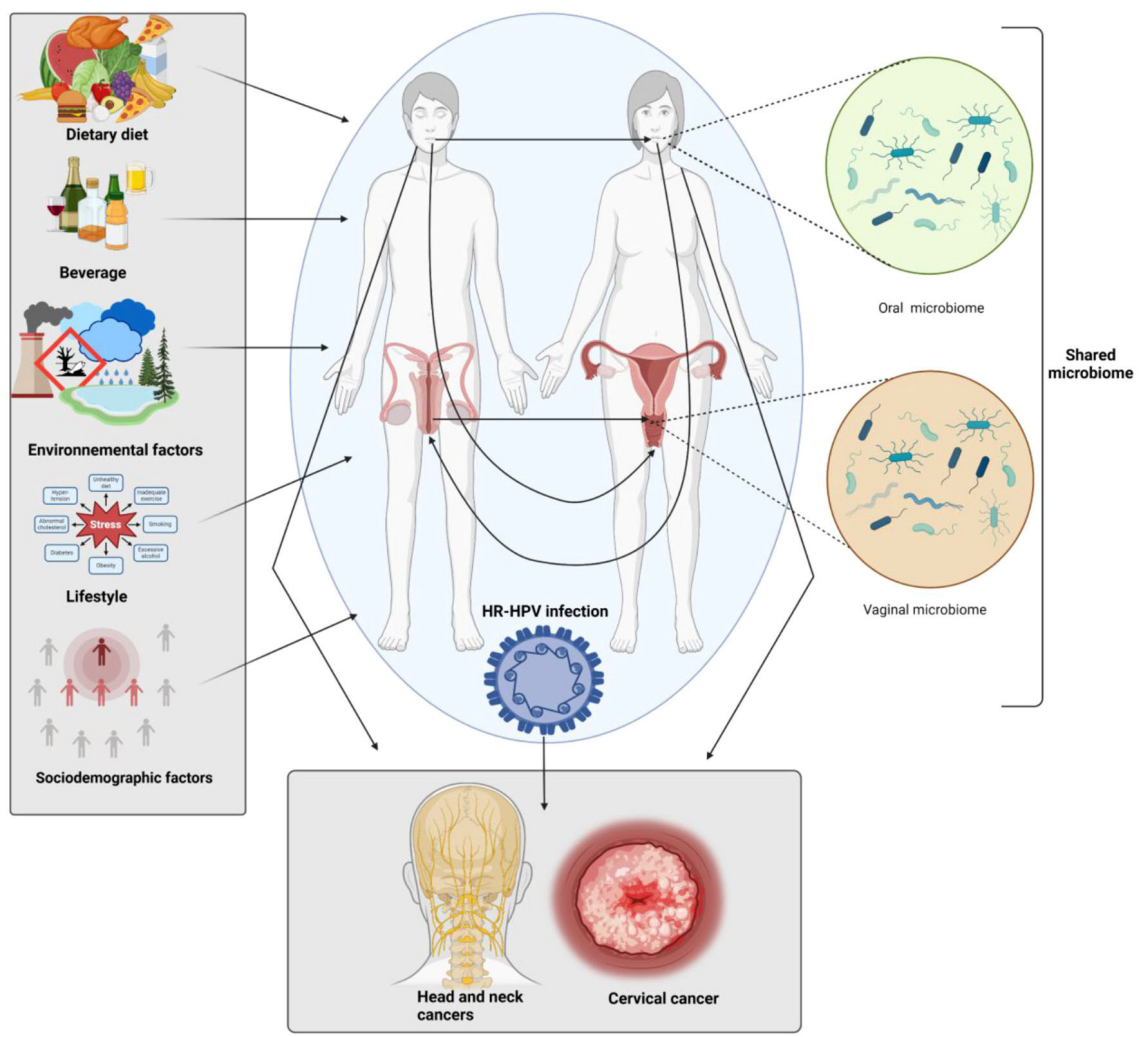
The Complex interplay between oral and vaginal microbiomes in high-risk HPV-induced cervical and head and neck cancers. External factors such as dietary habits, beverage consumption, environmental exposures, lifestyle choices, and sociodemographic characteristics, and their influence on both the oral and vaginal microbiomes. These factors are shown to affect microbiome composition, potentially leading to dysbiosis, a disruption of microbial balance. The shared oral and vaginal microbiomes are highlighted as key contributors to the development of HPV-associated cancers, particularly head and neck cancers and cervical cancer. Dysbiosis in either microbial community, driven by external or internal factors, may play a significant role in cancer progression. This figure emphasizes the importance of maintaining microbiome homeostasis as a potential strategy for the prevention and management of HPV-related.cancers. Generated in Biorender.com.

The oral microbiome refers to the community of microorganisms that naturally reside in the mouth. These microorganisms play a crucial role in maintaining oral health and overall well-being. The oral mucosa is second in size only to the gut in human microbial communities (Deo and Deshmukh, 2019). The two components of the human microbiome are the variable microbiome and the core microbiome. The core microbiome is shared among all individuals, whereas the variable microbiome is specific to each person, influenced by lifestyle, diet, socioenvironmental factors, and physiological distinctions (Zaura et al., 2014). However, certain sexual practices have been found to influence the oral microbiome and potentially impact the risk of developing head and neck cancer. In fact, current sexual practice includes oral sex, which involves the exchange of bodily fluids, including saliva and genital secretions. In modern heterosexual and homosexual relationships, oral sex is highly prevalent. Before, during, or after sexual activity, people may engage in oral sex as part of their foreplay (Saini et al., 2010). During oral sex, different species of microorganisms can be transmitted between partners, leading to changes in the oral microbiome (Maier, 2023). In addition, oral sexual practices facilitate the bidirectional exchange of microorganisms between the genital and oral mucosa (**Figure 3**). This microbial transfer can disrupt the ecological balance at either site, potentially converting commensal species into opportunistic pathogens or altering their functional roles within the new environment (Plummer et al., 2019). Such cross-site microbial exchange may therefore influence HPV transmission dynamics, mucosal immunity, and the progression of infection toward malignancy (Luo et al., 2024). Furthermore, oral-genital contact can facilitate the transmission of various sexually transmitted infections (STIs) such as herpes, gonorrhea, and the human immunodeficiency virus (HIV) (Gillison et al., 2000; Boekeloo and Howard, 2002). HPV specifically has been strongly associated with oropharyngeal cancer, a subtype of head and neck cancer. Studies have shown that individuals who engage in high-risk sexual behaviors or have multiple sexual partners may have a more diverse oral microbiome, including an increased prevalence of potentially harmful bacteria (Vodstrcil et al., 2017). For instance, a retrospective cross-sectional study comparing oral microbiota of women who are not engaged in sex work and women engaged in sex work, have shown that high-risk sexual behavior is associated with diversity of the oral microbiota and lack of *Lactobacillus* (Wessels et al., 2017). This alteration in the oral microbiome composition may contribute to the development of chronic inflammation and tissue damage in the oral cavity, predisposing individuals to head and neck cancer.

Mechanistically, Oral dysbiosis induces a persistent inflammatory milieu that elevates levels of cytokines and proteases, promotes oxidative stress, and undermines epithelial barrier integrity (Maier, 2023). Such chronic inflammation can promote HPV persistence by impairing effective antiviral responses and enhancing oncogenic processes by increasing DNA damage and cell proliferation (Senba and Mori, 2012). In addition, microbial metabolites (for example, short-chain fatty acids, nitrosamines, and other bioactive molecules) and bacterial enzyme activity can modulate local epithelial metabolism and epigenetic regulation, creating a microenvironment that favors viral replication and malignant transformation (Hanus et al., 2021). Biofilm formation and close bacterial–epithelial interactions may further protect virions from immune clearance and facilitate prolonged mucosal exposure to genotoxic factors (Vestby et al., 2020).

## 11 Cervicovaginal and oral microbiome dysbiosis: implication for cervical and head and neck cancers development

**Figure 4:**
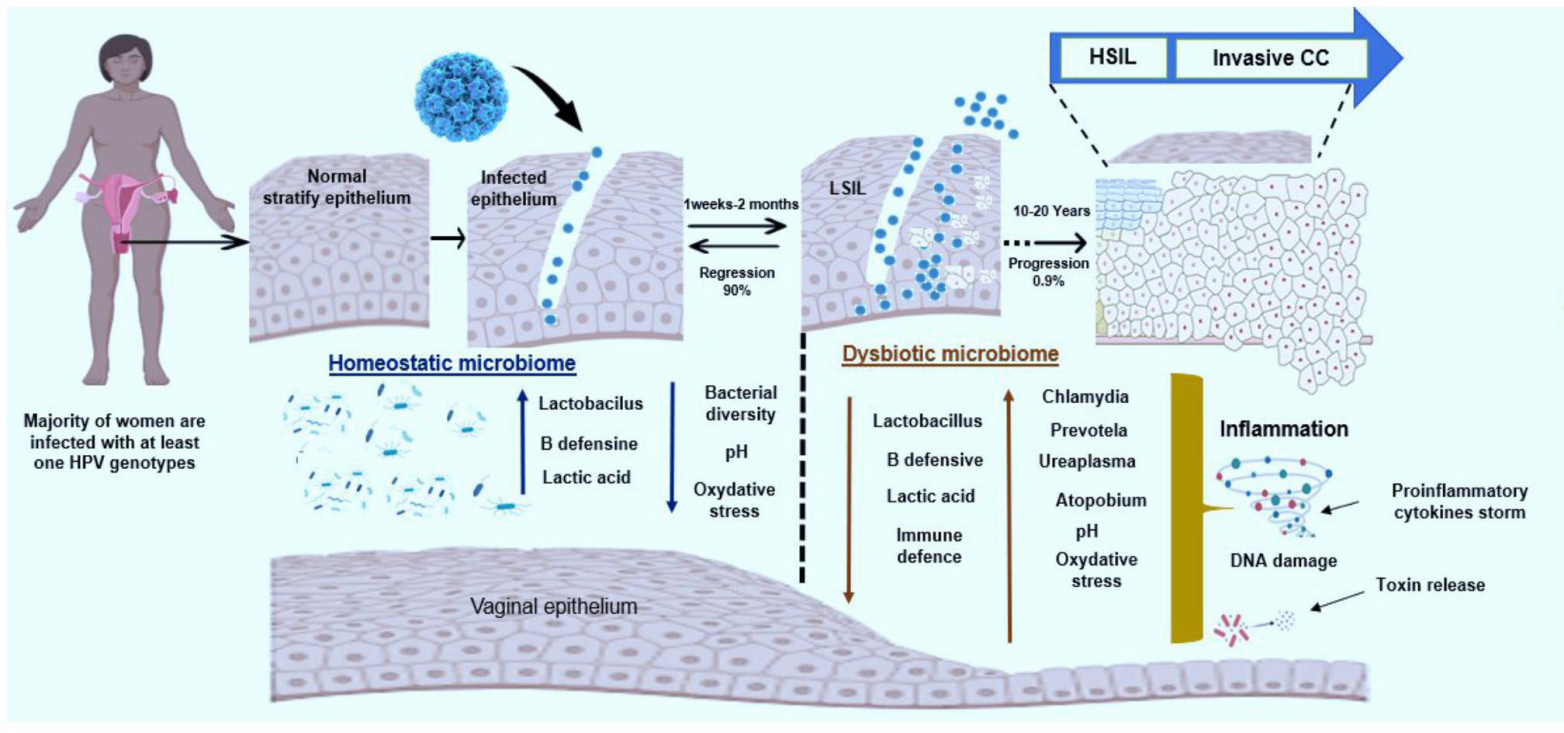
Proposed model of HPV infection, vaginal dysbiosis, and cervical cancer progression. Most women encounter HPV at least once in their lifetime. The normal cervical epithelium comprises stratified squamous cells that are resistant to viral infection. Microablation enables HPV entry and exposes the basal layer to the virus. HPV multiplies actively in immature epithelial cells, upon terminal differentiation, the virus is released. The repetition of this phenomenon induces Low-Grade Intraepithelial Lesions (LSIL) that can regress in the presence of normal vaginal microbiota. Altered vaginal microbiome combined with other co-factors induces sustained inflammation, DNA damage, and LSIL progression to High-Grad Intraepithelial Lesion (HSIL) and invasive Cervical Cancer (CC). Generated in Biorender.com.

The change in the natural vaginal microbiota can lead to several conditions including bacterial vaginosis (BV), vulvovaginal candidiasis (VC), Aerobic vaginitis (AV), Atrophic vaginitis (AV), and Atrophic vaginitis (Koumans et al., 2007; Sobel, 2007; Kaambo et al., 2018). BV is the most common vaginal disbalance in sexually active women. It’s characterized by a remarkable reduction of Lactobacilli and overgrowth of non-Lactobacilli microbes, such as anaerobic bacteria. BV has been associated with numerous reproductive health concerns including pelvic inflammatory disease, adverse obstetric outcomes, and diverse STI, including HIV and HPV (Kaambo et al., 2018). Vaginal dysbiosis usually affects immune homeostasis, inducing a rupture in the epithelial barrier and favoring STIs; thus, emerging evidence supports the hypothesis that the change in vaginal microbiome is involved in the natural history of several STIs including HPV (Lin et al., 2022). Several studies have suggested that vaginal dysbiosis may play a role in the progression of HPV infection to cervical cancer. For example, a disrupted vaginal microbiome can lead to chronic inflammation, which can promote growth and persistence of HPV-infected cells (Shen et al., 2024; Zhang et al., 2024). Additionally, changes in the vaginal environment, such as alterations in pH or production of toxic metabolites by certain bacteria, may also contribute to development of cervical dysplasia (Zhou et al., 2021). Furthermore, dysbiosis has been associated with a higher prevalence of HPV infection and a lower likelihood of spontaneous clearance of the virus (Mei et al., 2022). This suggests that maintaining a healthy vaginal microbiome may be important for preventing the progression of HPV infection to cervical cancer. Strategies to promote a healthy vaginal microbiome, such as probiotics or targeted antimicrobial therapies, may therefore have the potential to reduce the risk of cervical cancer in women with HPV infection (**Figure 4**).

It has been hypothesized that increased nitrosamine content produced by CST-IV which is not lactobacillus dominant, possibly results in higher DNA damage, change in immunopolarization and cytokine profile thus compromising immune defense against HPV infection (Kyrgiou et al., 2017). This suggestion might be relevant for a high number of CD4^+^ CD25^+^ regulatory T cells, as well as the activated Th2 cells associated with suppression of cytotoxic functions, reported in HR-HPV persistent infections (Kyrgiou et al., 2017). While vaginal dysbiosis has been extensively characterized as a cofactor in HPV persistence, growing evidence indicates that similar inflammatory and immunosuppressive patterns occur within the oral mucosa, suggesting shared mucosal mechanisms that may underlie HPV-driven tumorigenesis (Schellekens et al., 2025). In the vaginal niche, a shift from *Lactobacillus*-dominated communities to those enriched with anaerobic bacteria such as *Gardnerella vaginalis*, *Atopobium vaginae*, and *Prevotella* species has been linked to persistent HPV infection, epithelial disruption, and heightened local inflammation (Borgdorff et al., 2016; Sheng et al., 2025). Comparable microbial imbalances in the oral cavity marked by increased abundance of *Fusobacterium nucleatum*, *Porphyromonas gingivalis*, and *Candida albicans* have been associated with chronic mucosal inflammation and immune evasion, in the oral cavity of patients with HPV-positive head and neck cancers. both of which may facilitate viral persistence and malignant transformation (Liu et al., 2022; Moreno et al., 2023). These parallels indicate that dysbiosis-driven modulation of mucosal immunity is a common denominator across anatomical sites affected by HPV infection (Moreno et al., 2023). For instance, it was shown that patients with oral cancer harbor a virulent oral microbiome. *Fusobacterium nucleatum* is a Gram-negative anaerobic bacterium that was first isolated from the oral cavity and identified as a periodontal pathogen*. H*igher transcript levels of this bacteria have been reported in tumors and tumor-adjacent tissues of patients with oral squamous cell carcinoma (OSCC) compared to those of healthy controls (McIlvanna et al., 2021). Furthermore, *Porphyromonas gingivalis* has demonstrated the capacity to promote oncogenesis within the oral cavity. Research has revealed that these bacteria can stimulate the generation of myeloid-derived dendritic suppressor cells, which in turn inhibit cytotoxic T lymphocytes. Additionally, they induce overexpression of pro-matrix metalloproteinase-9 and decrease TP53 expression, thereby fostering cell proliferation, releasing genotoxins, generating carcinogens, inhibiting the synthesis of anticarcinogenic compounds, and creating a pro-tumor local microenvironment conducive to tumor growth (Biswas et al., 2022; Liang et al., 2022; Benjamin et al., 2023; Constantin et al., 2023). Collectively, these mechanisms play a role in malignancies, including HNCs (Constantin et al., 2023).

In addition, beyond bacterial communities, the role of the oral mycobiome, particularly *Candida* species, in HPV-related carcinogenesis has been reported. *Candida albicans* can induce epithelial hyperplasia, promote nitrosamine and acetaldehyde production, and stimulate pro-inflammatory cytokine expression, all of which create a microenvironment conducive to malignant transformation (Naglik et al., 2017; Di Cosola et al., 2021). For instance, it has been reported that *Candida albicans* infection impairs the metabolism of oral epithelial cells and downregulates cellular glycolysis (Zhao et al., 2024). Furthermore, *Candida*-induced inflammation leads to the release of reactive oxygen species (ROS) (Duan et al., 2025) and proinflammatory cytokines such as IL-6, IL-8, and TNF-α, which may synergize with HPV oncoprotein activity to enhance DNA damage leading to precancerous lesions and OSCC (G et al., 2025).

## 12 Factors influencing change in vaginal and oral microbiome in sub-Saharan Africa

The vaginal microbiome changes throughout a woman’s life, with the greatest variation occurring during puberty and menopause. In sub-Saharan Africa, the age of menarche is relatively early, which increases the risk of reproductive tract infections (RTIs) among young females. Hormonal changes during the menstrual cycle, pregnancy, and menopause affect the composition of the vaginal microbiome. These changes can cause the overgrowth of harmful bacteria, leading to RTIs. Sexual activity is also a crucial factor in shaping the va12ginal microbiome and increasing the risk of RTIs. In sub-Saharan Africa, cultural practices, such as early marriage and female genital mutilation, increase the risk of RTIs. Poor hygiene practices, such as the use of unclean materials during menstruation and female genital mutilation especially in SSA increase the risk of RTIs.

## 13 Methods used for microbiota profiling in Sub-Saharan Africa

Amsel criteria or Gram-staining Nugent score were formerly used in the majority of research describing the vaginal microbiota. Although these studies reveal that vaginal microbiota is commonly dominated by Lactobacillus bacterial species, their taxa classification were not possible (Barrientos-Durán et al., 2020). New culture-free techniques, such as new-generation sequencing have provided a deep insight into vaginal microbiota. Thus, Furthermore, in sub-Saharan Africa, especially in West Africa, most of the studies describing the vaginal microbiota used culture-based approaches, highlighting the need for molecular profiling of this crucial microenvironment. Furthermore, as the microbial profile varied according to specific populations, profiling the vaginal microbiota in sub-Saharan African women is critical to identify bacterial imbalances associated with cancer progression. There is a need to assess the microbial signature in every population to decipher the individuals at high risk of cervical cancer. Furthermore, the available data display a specific heterogeneity in study design, sample collection, and methodological approach (**Table 1**). CC, HPV, and microbiota characterization can help develop targeted interventions, such as probiotics or personalized treatment strategies to restore healthy vaginal microbiota and microbiome-based therapeutic for cervical cancer prevention toward its elimination as a public health concern.

**Table 1:** Summary of studies investigating the relationship between HPV infection, vaginal microbiome, and cervical cancer.

Country	Sampling strategy	HPV genotypes screened	Finding	NGS techniques	Reference
CHINA	- HPV-infected women without CIN (n=7) - Women with LSIL, n = 51 - Women with HSIL, n = 23 - Women with invasive cervical cancer (n=9) - healthy women without HPV infection (Normal, n = 68).	Not documented	- More richness and diversity in CC women. - Negative influence of HPV infection lactobacillus relative abundances. - the relative abundance of Prevotella, Bacillus, *Anaerococcus, Sneathia, Megasphaera*, *Streptococcu*s, and *Anaerococcus* in HPV infection.	16S rRNA gene amplicons (Illumina MiSeq)	(Chen et al., 2020)
Unites Kingdom	- LSIL (n = 52), - High-grade women (HSIL; n = 92) - Invasive cervical cancer (ICC; n = 5) - Healthy controls (n = 20).	HPV 16 HPV 18	- high-diversity and low levels of Lactobacillus - increasing disease severity, irrespective of HPV status - Increasing disease severity associated with decreasing relative abundance of Lactobacillus spp. - vaginal microbiome in HSIL characterized by higher levels of *Sneathia sanguinegens* (P < 0.01), *Anaerococcus tetradius* (P < 0.05), and *Peptostreptococcus anaerobius* (P < 0.05) and lower levels of *Lactobacillus jensenii* (P < 0.01) compared to LSIL.	16S rRNA gene amplicons sequencing (Illumina MiSeq)	(Mitra et al., 2015)
MEXICO	- SIL women HPV positive (n=121) - healthy women without HPV infection or SIL (n=107).	HPV 16	- SIL was associated with changes in beta diversity and higher species richness - HPV induces microbiota change irrespective of SIL - Independent associations between HPV infection and an increase in relative abundance of *Brachybacterium conglomeratum, Brevibacterium aureum* and decrease in two Lactobacillus iners OTUs. - Positive independent association between HPV-16 and *Brachybacterium conglomeratum*.	16S rRNA gene amplicons (Illumina MiSeq)	(Nieves-Ramírez et al., 2021)
CANADA	- 59 participants as follows: 36 HPV negative 23 HPV positive	37 genotypes including HR-HPV (16, 18, 31, 33, 35, 39, 45, 51, 52, 56, 58, 59, 68 and 69.	- High correlation between CST distribution and BV status, - HPV positivity correlated with high diversity of cervico-vaginal microbiome (CST-IV) and less RA of *L. gasseri.*	16S rRNA gene amplicons (Illumina MiSeq)	(Shannon et al., 2017)
POLAND	- 16 patients with SCCC - 30 healthy women	NA	- Significant alterations in the CM of cervical cancer patients compared to healthy controls. - Heterogenous CM community in the cancer groups.	16S rRNA gene amplicons (Ion Torrent)	(Zeber-Lubecka et al., 2022)
ROMANIA	- LSIL (n=18) - ASCUS (n=16) - ASCH (n=13) - SCC (*n* = 9) - NILM (n=20) including: HPV+ (n=20) HPV- (n=20)	13 HR-HPV: 16, 18, 31, 33, 35, 39, 45, 51, 52, 56, 58, 59, and 68.	- Significant association between microbiota diversity, HPV infection, and cervical lesion progression - *Presence of Lactobacillus iners* aand absence of *Lactobacillus crispatus, Atopobium* spp., *Prevotella* spp., and *Gardnerella* spp associated with severe cervical lesions.	16S rRNA gene amplicons (Ilumina)	(Stoian et al., 2023)
SOUTH AFRICA	- 87 participants including: 37 LR-HPV 30 HR-HPV 20 healthy women	37 HPV types (13 HR and 24 low-risk).	- Majority of participants (74%) had cervical microbiota not dominated by Lactobacillus. - Lactobacillus was not enriched in HPV-negative women compared to HPV-positive women. - No correlation between cervical microbiota diversity and HPV infection.	16S rRNA gene amplicons (Illumina MiSeq)	(Onywera et al., 2019)
USA (Arizona)	- HPV-negative (n = 20) - HPV-positive (n = 31) - LGD (n = 12) - HGD (n = 27) - Invasive cervical carcinoma (n = 10)	37 genotypes including HR-HPV (16, 18, 31, 33, 35, 39, 45, 51, 52, 56, 58, 59, 68 and 69.	- *Lactobacillus* dominance decreased with the severity of cervical neoplasm - *Sneathia* was enriched in all precancerous groups	16S rRNA gene amplicons (Illumina MiSeq)	(Łaniewski et al., 2018)
BOTWANA	- 31 patients: Dysplasia (21) Cancer (10)	NA	- Cervical microbiome diversity was greater for cancer versus dysplasia - Cervical microbiota of women with cancer are distinct in composition as compared with dysplasia	16S rRNA gene amplicons (Illumina MiSeq)	(Sims et al., 2020)

NA, Not applicable; SIL, Precancerous squamous intraepithelial lesion; CIN, Cervical Intraepithelial Lesion; OTUs, operational taxonomic units; CST, community state type; RA, Relative abundance; HR-HPV, High-Risk HPV; LR-HPV, Low-Risk HPV; SCCC, Squamous Cell Carcinoma of the Cervix; CM, Cervical microbiome; ASCUS, Atypical Squamous Cells of Undetermined Significance; ASCH, Atypical Squamous Cells; SCC, squamous cervical carcinomas; NILM, Negative for Intraepithelial Lesion or Malignancy; HGD, High-grade dysplasia; LGD, Low-grade dysplasia; ICC, invasive cervical carcinoma.

## 14 Concluding remark

Oral and vaginal dysbiosis are emerging risk factors in the progression of cervical and head and neck cancers, respectively. The unique challenges faced by sub-Saharan Africa highlight the importance of studying the oral and vaginal microbiota and its role in cancer development and progression. Further research is needed to improve cancer prevention, early diagnosis, and treatment strategies in SSA. The distinct characteristics of vaginal microbiota in sub-Saharan African women compared to women in other regions necessitate further research to understand the role of dysbiosis in cancer progression. Investigations on microbiome dysbiosis in Sub-Saharan Africa (SSA) show that tailored microbiota profiling can significantly improve cancer prevention and treatment efforts. By identifying particular microbial signatures linked to higher cancer risk, healthcare practitioners may create individualized screening methods and preventative actions that are culturally and contextually appropriate for the location. Furthermore, implementing preventative measures focused at restoring a healthy microbiome, such as probiotic usage and dietary changes, has the potential to greatly lower the incidence of cervical, head, and neck cancers, especially in SSA.

Integrating microbiota assessments into cancer care allows for the tailoring of therapies to individual patients, thereby improving treatment outcomes and minimizing adverse effects. This personalized approach not only addresses the immediate needs of patients but also contributes to a broader understanding of how microbiome health impacts cancer progression. In addition to these clinical applications, raising public awareness about the relationship between microbiome health and cancer risk is essential. Educational initiatives can empower communities to adopt healthier lifestyle practices, which may further mitigate cancer risk. Fostering interdisciplinary collaborations between microbiologists, oncologists, and public health experts can lead to innovative research and practical solutions that address the complexities of cancer in SSA.

Despite the growing recognition of the microbiome’s role in cancer prevention and management, routine implementation of sequencing-based microbiome profiling in clinical settings remains a major challenge in sub-Saharan Africa due to economic and infrastructural limitations. High-throughput sequencing technologies, though powerful, are often costly, require specialized equipment, and depend on trained personnel and stable supply chains all of which are scarce in many healthcare facilities across the region. Consequently, most cancer diagnostic centers lack the capacity to perform such advanced analyses, restricting microbiome research and its translation into clinical practice. Addressing these barriers will require the development of affordable, rapid, and context-appropriate diagnostic tools, as well as investments in local capacity building and laboratory infrastructure to ensure that microbiome-based cancer prevention strategies are both sustainable and accessible within resource-limited settings.

## 15 Strengths and limitations

This review provides an integrative overview of the interplay between HPV infection, immune evasion, and microbiome dysbiosis, emphasizing their relevance within the sub-Saharan African context. By addressing both vaginal and oral microbial ecosystems, it offers a novel, cross-mucosal perspective on HPV-driven carcinogenesis and highlights the potential of microbiota profiling in improving disease prevention and management strategies in resource-limited settings. Nonetheless, the lack of a systematic literature selection process and the scarcity of region-specific molecular data may limit the generalizability of the findings. Future investigations should incorporate host genetic, environmental, and lifestyle determinants to better elucidate population-specific HPV–microbiome interactions.
